# A Comparison Between Ripening Under a Constant Volume and Ripening Under a Constant Surface Area

**DOI:** 10.3390/nano15040316

**Published:** 2025-02-19

**Authors:** King-Ning Tu, Andriy M. Gusak, Qinglei Sun, Yifan Yao

**Affiliations:** 1 Department of Materials Science and Engineering, City University of Hong Kong, Kowloon, Hong Kongyifanyao3-c@my.cityu.edu.hk (Y.Y.); 2 Department of Electrical Engineering, City University of Hong Kong, Kowloon, Hong Kong; 3 Department of Physics, Cherkasy National University, 81 Shevchenko Blvd., 18000 Cherkasy, Ukraine; amgusak@ukr.net; 4 Ensemble3 Centre of Excellence, 133 Wolzcynska Street, 01-919 Warsaw, Poland; 5 Institute for Advanced Marine Research, China University of Geosciences, Guangzhou 511462, China

**Keywords:** ripening, phase transformation, electromigration, thermomigration

## Abstract

The classic Lifshitz–Slyozov–Wagner (LSW) theory of ripening assumes a constant volume. In comparison, we present here a model of ripening assuming a constant surface area, which has occurred in the microstructure changes in intermetallic compounds in micro-bump for 3D integrated-circuit (IC) technology in consumer electronic products. However, to keep a constant surface area requires the growth of the volume. Furthermore, in 3D IC technology, the kinetics is affected by electrical charges flowing in and out of the system. Due to Joule heating and electromigration, heat flux and atomic flux can occur together. The kinetic modes of failure changes are given here, as well as the mean-time-to-failure equations based on entropy production.

By definition, a flux of moving atoms has to be driven by a chemical potential gradient. Typically, we consider the temperature gradient in thermomigration, pressure (stress) gradient in stress migration, as well as electrical potential gradient in electromigration. In electromigration, atoms will diffuse along the electron flow direction, leading to atomic loss at the cathode end, while intermetallic compound (IMC) formation at the anode end. As a result, the resistance of the interconnect increases. The industrial field usually defines the resistance increases of 20% as an electromigration failure criterion. Furthermore, volume shrinkage occurs when IMCs form, resulting in crack/void formation. However, electromigration occurs in open systems because of the in-and-out of charges. On the other hand, in a classical phase transformation, such as precipitation, it occurs in a closed system [1,2,3,4,5]. The rate process in a classical phase transformation can be diffusion-limited or interfacial-reaction-limited. However, in an open system, it is typically supply-limited [6].

Many papers have investigated the ripening phenomenon [7,8]. The first literature on ripening in flux-driven reaction (FDR) was about the reaction between liquid-state eutectic SnPb and solid-state Cu to form the scallop-type IMC of Cu6Sn5 after reflow for 1 min at 200 °C. The liquid channel between two scallops provides Cu atoms with rapid diffusion paths, leading to the ripening of the scallop-type Cu6Sn5. Therefore, it is a flux-driven ripening. Figure 1 shows the cross-sectional view of semi-spherical scallops in a circular solder cap on a Cu surface. There is a deep channel between two scallops. These channels are not grain boundaries, and they allow Cu to diffuse rapidly to the solder to grow the scallops by ripening.

What is unique in the above figure is that the scallops do not become a “diffusion barrier” to the subsequent growth of scallops. This is unlike the layer-type growth of an IMC, which will become a diffusion-barrier to its subsequent growth. We note that, in the manufacturing of 3D IC technology, several reflows of solder joints are required. During each reflow, the growth of scallops continues to occur by ripening. Therefore, the scallop-type growth of IMCs is essential in solder joint technology.

A critically important parameter, which controls the kinetics of ripening of scallops, is that the total surface area of all the scallops remains constant, which is equal to twice of the bottom contact area of the solder cap. In the classical theory of Lifshitz–Slyozov–Wagner (LSW) ripening, the total volume of all the precipitates is assumed to be constant. In the ripening of solder scallops, the total surface area of all the scallops, assuming a hemispherical shape, is constant, which is twice of the contact area, or it is equal to the surface area of the largest half sphere. The theory of non-conservative ripening with a constant surface area has been published elsewhere [6]. Here, we make a comparison between the ripening under a constant volume and the ripening under a constant surface area.

Many studies on electromigration have been conducted to find out its failure mechanism or predict its lifetime [9,10,11]. Now, we consider electrical conduction of a pure metal wire under a constant temperature and a constant pressure. We obtain *TdS* = *dU*, from the first law of thermodynamics, where the unit of electric energy “*dU*” is “eV” or charge times voltage. In the equation below, *jAdt* is the charge, Δφ is the voltage drop, and Δ*x* is the size of the interconnect where electrons flow.

(1) $$TdS=dU=jAdt\Delta \varphi =jAdt[\varphi (x)-\varphi (x+\Delta x)]=-jVdt[\frac{\varphi (x+\Delta x)-\varphi (x)}{\Delta x}]=jVdt[-\frac{d\varphi }{dx}]$$

(2) $$\frac{TdS}{Vdt}=j[-\frac{d\varphi }{dx}]=jE=j^{2}\rho$$

where *dS*/*dt* is rate of entropy production, *T* is absolute temperature, *V* is volume of the test sample, *j* is current density, *Φ* is voltage, *E* is electric field, and *ρ* is resistivity.

In the above, we obtained the power of Joule heating (*P* = *I*^2^*R* = *j*^2^*ρV*, where *I* is current and *R* is resistance). This is also Onsager’s equation of entropy production. Onsager defined the conjugate forces *X* and fluxes *J* so that their product, *JX*, is equal to temperature *T*, multiplied by the entropy production rate, “*dS*/*dt*”.

(3) $$\frac{T}{V}\frac{dS}{dt}=JX$$

where *V* is volume. By rearrangement, we have in electromigration,

(4) $$J_{e}X_{e}t^{failure}=TS_{threshold}/V$$

We treat MTTF as the time to accumulate some threshold entropy, *S_threshold_*. Therefore, for electromigration, its mean-time-to-failure (MTTF) equation is given below [12],

(5) $$MTTF=Aj^{-n}exp(\frac{E_{a}}{kT})=t^{failure}=\frac{TS_{threshold}}{VJ_{e}X_{e}}=A\frac{1}{j^{2}}\frac{1}{D}=Aj^{-2}\exp(\frac{E_{a}}{kT})$$

where *A* is pre-factor, *n* is the current density power factor, *D* is diffusivity, *k* is the Boltzmann constant, and *E_a_* is activation energy. Owing to the fact that in the above equation, n = 2 has been derived theoretically, furthermore, the activation energy (Ea) in a typical material, such as solder joint, is known, so that the only one unknown parameter in the above equation is the pre-factor A, which means that we will only need to perform 1T1j experiment to determine it. This means that we have greatly simplified the experimental study of the MTTF equation.

Table 1 below shows a direct comparison between the two processes. In constant volume ripening in a closed system, the total surface area decreases as ripening occurs; for example, in the joining of two rain drops under a wire, it is the reduction in surface energy that drives the phase transformation. However, in constant surface area ripening in an open system, the total volume increases, which is driven by the increase in bulk free energy.

In summary, examples of flux-driven phase transformations in an open system, with electrical charges flowing in and out, have been presented. What is new in flux-driven phase transformations, as compared to the classical phase transformations, is in the kinetic processes rather than in the driving forces. While we have atomic flux, heat flux, and charge flux, it is their cross-effects which tend to have significance on the reliability of micro-electronic devices. What is worth mentioning is that, in the flux-driven growth of scallop-type Cu-Sn intermetallic compounds during solder joint formation, its growth does not become a diffusion barrier to its subsequent growth. It enables us to apply multiple reflows of solder joints, which is required in the manufacturing of advanced packaging technology. Finally, it is worth mentioning again that, in LSW theory of ripening, the total volume is assumed to be constant. However, in the ripening theory presented here, as shown in Ref. [6], the total surface area is assumed to be constant.

## Figures and Tables

**Figure 1:**
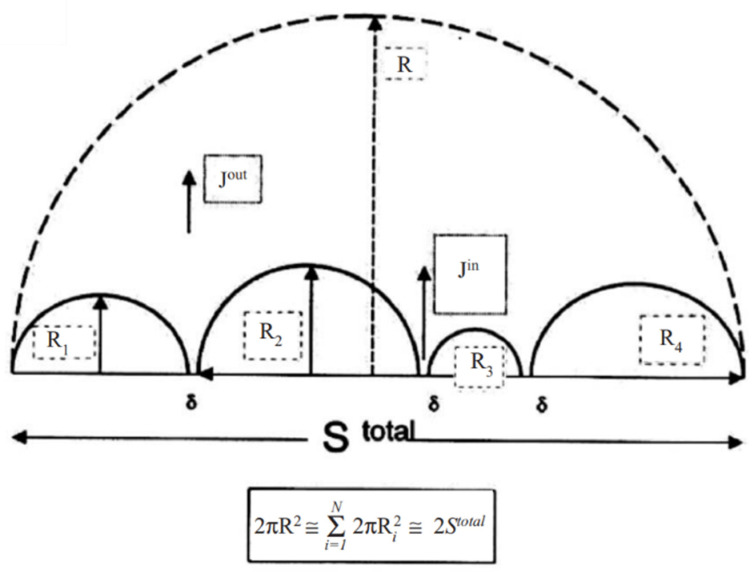
The cross-sectional view of semi-spherical scallops in a circular solder cap on a Cu surface.

**Table 1:** A comparison between constant volume ripening and constant surface area ripening.

	Constant Volume Ripening	Constant Surface Area Ripening
Total Volume	Constant	Increase
Total Surface Area	Increase	Constant
